# Research on “The Belt and Road Initiative” report of think tank based on theme evolution and identification——Taking 2013–2020 as an example

**DOI:** 10.1371/journal.pone.0297127

**Published:** 2024-06-18

**Authors:** Hao FU, Feng WEI, Hong ZHOU, A-mei DENG

**Affiliations:** 1 National Science Library (Wuhan), Chinese Academy of Sciences, Wuhan, Hubei, China; 2 Department of Information Resources Management, School of Economics and Management, University of Chinese Academy of Sciences, Beijing, China; 3 Hubei Key Laboratory of Big Data in Science and Technology, Wuhan, Hubei, China; Hosei University: Hosei Daigaku, JAPAN

## Abstract

China’s "the Belt and Road Initiative" (BRI) is a top-level cooperation initiative among countries proposed by China, which has promoted China’s cooperation with relevant countries in various aspects and fields. Research reports from think tanks and experts on the evaluation, analysis, and research conclusions of the BRI can reflect the stance, opinions, and demands of various countries abroad regarding the initiative. This paper takes the BRI reports of important think tanks in the " Global Go To Think Tank Index Report 2020" as the subject of its research, and analyzes the key points and development trends of foreign think tank research on the BRI by using text mining, topic evolution, and social network analysis. It provides reasonable suggestions and ideas for promoting the construction of the BRI and deepening related cooperation in China. Research shows that the thematic distribution of research reports on the BRI by think tanks is mainly focused on the fields of politics, economy, and military. The research areas are relatively stable, and there is not a strong trend of thematic evolution. The evolution paths are also mainly distributed in the fields of politics, economy, and military. There are not many expansions in the thematic evolution directions over the years, and there is a strong inheritance of themes. The connection between research themes and the main purpose of the BRI is somewhat inadequate, indicating a certain limitation in the understanding of the BRI.

## Introduction

The Belt and Road Initiative (BRI) is a national top-level cooperative initiative proposed by Chinese President Xi Jinping in 2013. The proposal has attracted close attention and positive response from countries around the world. BRI has promoted mutually beneficial cooperation among many countries in Asia, Europe, and Africa, and has also achieved friendly exchanges between China and the international community in the fields of economy, politics, culture, and more. The research achievements of think tanks have unique cutting-edge and guiding significance in analyzing, evaluating, and guiding national construction. They are important references and information sources for government strategic planning, corporate strength enhancement, and public opinion understanding. They have important implications for the development of the country’s economy, politics, science and technology, and other aspects. In the development process of the BRI, think tanks from China and foreign countries serve as important actors in public diplomacy. Through their professional analysis and scientific research, they provide high-quality guidance, evaluation, and reference for the development of the BRI. Studying research reports on the BRI by think tanks can reveal the key areas of international attention towards the initiative, promote efficient and scientific implementation of related work, and make it more targeted. Based on this, this article focuses on the research reports on the BRI by key think tanks in the "Global Go To Think Tank Index Report 2020" published by the Think Tanks and Civil Societies Program (TTCSP) at the University of Pennsylvania’s Lauder Institute. By using text information processing methods such as topic mining and topic evolution analysis, it analyzes the hot topics and trends in think tanks’ research on the BRI, providing a basis and reference for the advancement of the BRI strategy and related think tank research.

### Research preparation

The more rigorous think tank comprehensive evaluation index system in the " Global Go To Think Tank Index Report 2020" allows the think tanks listed in the " Global Go To Think Tank Index Report 2020" to have strong persuasiveness and representativeness. This article selects the top ten think tanks in the comprehensive ranking of the " Global Go To Think Tank Index Report 2020" as the key think tank samples, and crawls various materials such as BRI related research reports, expert opinions, and consulting analyses published on their official websites. The website retrieval keywords are "Belt and Road", "B&R", "BRI". After manual screening and deduplication, a total of 703 relevant think tank reports were crawled, with a total text length of 320,715 words. The think tank information and data are shown in the following Table 1.

**Table 1:** Data table of the BRI report of think tank "Global Go To Think Tank Index Report 2020".

No.	Think tank name	Time of establishment(year)	Report number of BRI	Text number of words
1	Carnegie Endowment for International Peace (United States)	1910	168	11276
2	Bruegel Institute (Belgium)	2005	60	6839
3	Giorgio Vargas Foundation (Brazil)	1944	90	32960
4	International Center for Strategic Research (USA)	1962	127	18630
5	Institute of International Relations (France)	1990	36	25773
6	Royal Institute of International Affairs (UK)	1920	25	14329
7	RAND Corporation (USA)	1948	86	44036
8	Japan Institute of International Studies (Japan)	1959	70	81060
9	Peterson Institute of International Economics (USA)	1981	25	42347
10	Woodrow Wilson International Scholars Center (USA)	1968	26	43465

To preprocess the text of think tank reports in English, including word segmentation, establishing a stop word dictionary, removing stop words, and part-of-speech tagging, to ensure compatibility with the research methods and experimental models of this article, and to provide a foundation for the subsequent analysis of topic mining and topic evolution research.

### Research on the BRI report of think tank based on theme mining

LDA topic model is currently the most widely used topic model, which has better performance in extracting document topics with strong interpretability. Word2vec word vector representation method trains word vectors through Skip-gram and CBOW models, and it has been widely used to improve the effectiveness of text representation. LDA2Vec model is a deep learning-based topic model proposed by Chris Moody and others in 2016. The main idea is to combine LDA topic model with word2vec word vector model to capture topics.

The reason for choosing LDA2Vec in this study is that it combines the advantages of LDA and Word2Vec. LDA2Vec combines the topic modeling ability of LDA and the word embedding technique of Word2Vec. This means that it can not only understand the overall thematic structure of the document, but also understand the subtle semantic differences of vocabulary. By learning word embeddings, LDA2Vec is able to capture the similarity and relationship between words, which is important for understanding implicit meanings and subtle differences in language. Additionally, the LDA2Vec model can work at different levels (word level and document level), providing a more comprehensive text analysis. By combining word embeddings and topic modeling, LDA2Vec can provide richer feature representations, thus improving document clustering and classification tasks. Compared to purely neural network models, LDA2Vec offers better interpretability, as it maps documents to explicit topics that are often interpretable. Finally, LDA2Vec effectively handles complex documents with multiple topics by combining topic modeling and word embedding, making it more suitable for the research at hand.

In this chapter, the preprocessed texts from 2013 to 2020 are organized and summarized based on the time window. The LDA2Vec model is then employed for iterative training to obtain the topics and associated keywords of the high-end think tank research reports on the BRI. Through multiple rounds of experiments and consideration of topic relevance, the number of topics for each year’s texts is determined based on the lowest perplexity value of the model. The number of keywords for each topic is uniformly set to 10, and the model is iterated 25 times. The topic mining results show no missing or redundant information, and the clustering effect of the topics is optimal. The mining results of the think tank reports on the BRI are presented in Table 2.

**Table 2:** The theme excavation results of the BRI report of think tanks in Global Go To Think Tank Report 2020.

Time(year)	Theme	keywords
2013	regional influence and threat	Europe, country, Asia, western, developing, central, regional, influence, crisis, role
	regional trade and partnership	relations, central, influence, trade, society, strategic, partnership, economic, political, cooperation
2014	military power and international security	regional, relations, country, development, Middle-East, border, organization, security, military, force
2015	infrastructure and investment	Initiative, cooperation, infrastructure, development, investment, trade, support, strategic, government, foreign
	market competition and economic status	Europe, western, crisis, political, debt, economy, relationship, war, market, financial
2016	political relations and regional development	relations, region, cooperation, political, development, economic, country, initiative, regional, policy
	infrastructure investment and capital transport	Pacific, trade, Asia, development, trade, infrastructure, investment, trans, interventions, cost
	political and economic leadership and Finance	banks, brotherhood, financial, growth, international, growth, cooperation, leadership, political, government
2017	international status and influence	cooperation, Asia, development, trade, issues, cooperation, international, influence, governance, administration
	infrastructure construction and economic trade	trade, development, economic, investment, infrastructure, summit, international, construction, strategic, support
2018	infrastructure construction and political status	projects, development, infrastructure, construction, investment, economic, America, political, positions, Asia
	Japanese relations and economic competition	APEC, trade, japan, pacific, economic, war, strategic, political, investment, positions
	international organization cooperation and leadership	Japan, pacific, Asia, APEC, ASEAN, sovereignty, influence, relations, administration, global
2019	energy transportation and trade	chine, pour, production, oil, energy, region, development, port, investment, studies
	global product market and infrastructure investment	development, projects, infrastructure, global, investment, production, companies, market, energy, economic
	U.S.-Japan alliance and tariff agreement	United States, Japan, agreement, union, administration, government, relations, political, tariffs, national
	global trade and economic project	trade, global, strategy, WTO, agreement, trade, economic, investment, issues, market
2020	infrastructure construction and partnership	partners, development, leadership, allies, investment, partners, infrastructure, financial, cooperation, private
	domestic growth and critical challenges	domestic, allies, change, critical, domestic, influence, growing, economic, impact, challenge
	humanitarian assistance and military forces	spending, aid, defense, humanitarian, assistance, security, military, control, warfare, commentary
	bilateral cooperation and economic ties	official, debt, bilateral, development, financial, cooperation, economic, ties, project, challenges
	political cooperation and legal assistance	corporation, rand, belt, development, commentary, law, approach, system, assistance, political

According to Table 2, the research focus of high-end think tanks on the BRI is as follows: in the initial stage from 2013 to 2017, the main research areas are politics, economy, military, and other fields. In 2013, when the BRI was just proposed, the research focus of think tanks was concentrated in the fields of politics and economy. This includes the development and impact of China-Europe, China-US, and China-Russia relations after the proposal of the BRI. It is believed that the BRI is a regional strategic initiative that emphasizes partnership. This is in line with the development of Asian regional integration and the impact manifested by third regionalism [1]. It is worth noting that the hot topics also mention the "crisis" sense brought to some countries by the proposal of the BRI. In 2014, the impact of the BRI in the Middle East became a hot topic, as did the military capabilities of the countries along the BRI and the international security issues involved, which became the focus of research that year. In 2015, the research focus returned to the attention on national relations, such as China-India, China-Europe, and China-US relations, with studies on political influence having a geopolitical nature [2], and emphasis on market research in the economic field. It highlights the significant role of regionalization and geopolitics in think tank research. In 2016, the economic field involved infrastructure, banking, investment, and the political field continued to analyze the impact of the BRI in Europe, Asia, and the Pacific region. In recent years, there has been growing attention in the international community towards the leadership effectiveness of the BRI, which started to gain prominence in 2017. Apart from examining the cooperative relations between governments, there is also a focus on issues such as economic investment and trade.

From 2018 to 2020, the research on the BRI by think tanks was in a developmental stage, with a diverse range of research topics. In 2018, the research scope covered the combined effects of the BRI with APEC and ASEAN organizations, as well as the study of Sino-Japanese relations within the framework of the BRI. In 2019, the research focus has expanded to include energy issues, particularly the trade of oil resources. This involves various economic aspects such as trade tariffs, product circulation, and capital investment. In addition, there is political attention towards the relationship between Europe and Asia, as well as the political effects of the BRI in Africa. In 2020, the main research hotspots were manifested in the areas of economic trade, political influence, and military capabilities. This includes the bilateral relations between China and countries along the BRI, economic cooperation, infrastructure construction and investment, food distribution, policy formulation, as well as legal issues.

From an overall perspective, major think tanks have primarily focused on political and economic research regarding the BRI, particularly on political influence and economic investment. This reflects the significant economic potential of international cooperation and investment attraction [3] associated with the BRI.

### Research on the BRI report of the think tank based on the theme evolution

In this section, the results of topic mining for the BRI research conducted by think tanks are arranged in chronological order. Semantic similarity calculations are performed between the topic words within each year’s theme. Specifically, the vectors of the topic words are mapped onto the word2Vec space and cosine similarity is calculated. First, it is necessary to obtain word embeddings. This can be achieved through a pre-trained word embedding model, such as Word2Vec. These models map each word to a vector in a high-dimensional space. With the vectors of two words, compute their dot product. The dot product is the sum of the element-wise multiplication of the two vectors. Next, calculate the norm (or length) of each vector. The norm of a vector is the square root of the sum of the squares of its elements. Finally, calculate the cosine similarity using the following formula:

$$\text{If}\;\text{A}\;\text{and}\;\text{B}\;\text{are}\;\text{two}\;\text{vectors},\;\text{the}\;\text{cosine}\;\text{similarity}=\frac{A\cdot B}{\left\|A\right\|\cdot \left\|B\right\|}$$ (1)

This value will range between -1 (completely opposite) and 1 (completely identical), with 0 indicating no correlation between the two vectors.To address the issue of negative values in the calculation results and to control the range of the results between 0 and 1, the method used for semantic similarity calculation is the binary cosine distance method (Cosine_distance/2), as shown in Formula 1:

Cosine_distance/2=(1−Cosine_similarity)/2 (2)

The more similar the themes are, the smaller the binary cosine distance, indicating that the calculation between samples is more similar. When the samples are completely identical, the binary cosine distance is 0, otherwise it is 1. This article takes the comparison table of think tank annual reports from 2016 to 2019 as an example, as shown in Table 3. The letter "t" represents the topic, and the number after "t" corresponds to each different topic. The similarity calculation results for some topics are displayed, and the partial results of semantic similarity calculation for the BRI report topic of think tanks are shown in Table 4.

**Table 3:** Comparison of Some Themes in the Report of Think Tanks on the BRI.

2016	2017	2018	2019
t1:political relations and regional development	t4:international status and influence	t6:infrastructure construction and political status	t9:energy transportation and trade
t2:infrastructure investment and capital transport	t5:infrastructure construction and economic trade	t7:Japanese relations and economic competition	t10:global product market and infrastructure investment
t3:political and economic leadership and Finance		t8:international organization cooperation and leadership	t11:U.S.-Japan alliance and tariff agreement
			t12:global trade and economic project

**Table 4:** Partial Results of Semantic Similarity Calculation of the Theme of Think Tank’s BRI Report.

2016–2017		2017–2018		2018–2019	
**Theme**	**Similarity**	**Theme**	**Similarity**	**Theme**	**Similarity**
t1;t4	0.05305	t4;t6	0.03485	t6;t9	0.07125
t1;t5	0.05330	t4;t7	0.05090	t6;t10	0.05880
t2;t4	0.05290	t4;t8	0.04530	t6;t11	0.04795
t2;t5	0.04455	t5;t6	0.03580	t6;t12	0.04960
t3;t4	0.05395	t5;t7	0.05290	t7;t9	0.09670
t3;t5	0.04380	t5;t8	0.04850	t7;t10	0.08510
				t7;t11	0.07180
				t7;t12	0.05850
				t8;t9	0.10385
				t8;t10	0.07630
				t8;t11	0.05790
				t8;t12	0.05970

Based on the semantic similarity calculation results based on Tables 3 and 4, it can be seen that from 2016 to 2019, the thematic similarity of think tanks on the BRI topic is generally between (0, 0.2), indicating a high degree of thematic similarity in the research on the BRI by think tanks from 2016 to 2019. The relationships between various research topics are close, and the differences between the research topics in each year are small.

In order to study the trend of theme evolution in a more refined manner, and to facilitate the exploration of theme split, theme fusion, theme continuation, and other theme evolution characteristics between each year, the average value of the similarity calculation results of each year is taken as an indicator to evaluate the evolution relationship between themes. For example, if the similarity calculation result between theme 1 in 2016 and theme 1 in 2017 is higher than the average value of semantic similarity calculation results between 2016 and 2017 themes, it can be considered that theme 1 in 2016 is more similar to theme 1 in 2017, and the theme relationship is closer, indicating finer features in theme evolution.

Based on the results of semantic similarity calculation and the average value indicator, summarize the evolution relationship of themes between each year. Utilize the Pyecharts tool for visualization and obtain the evolution trend graph of the research themes on the BRI by think tanks. Analyze the evolution characteristics of research themes on the BRI by domestic and think tanks based on the themes reflected in the graph.

**Fig 1:**
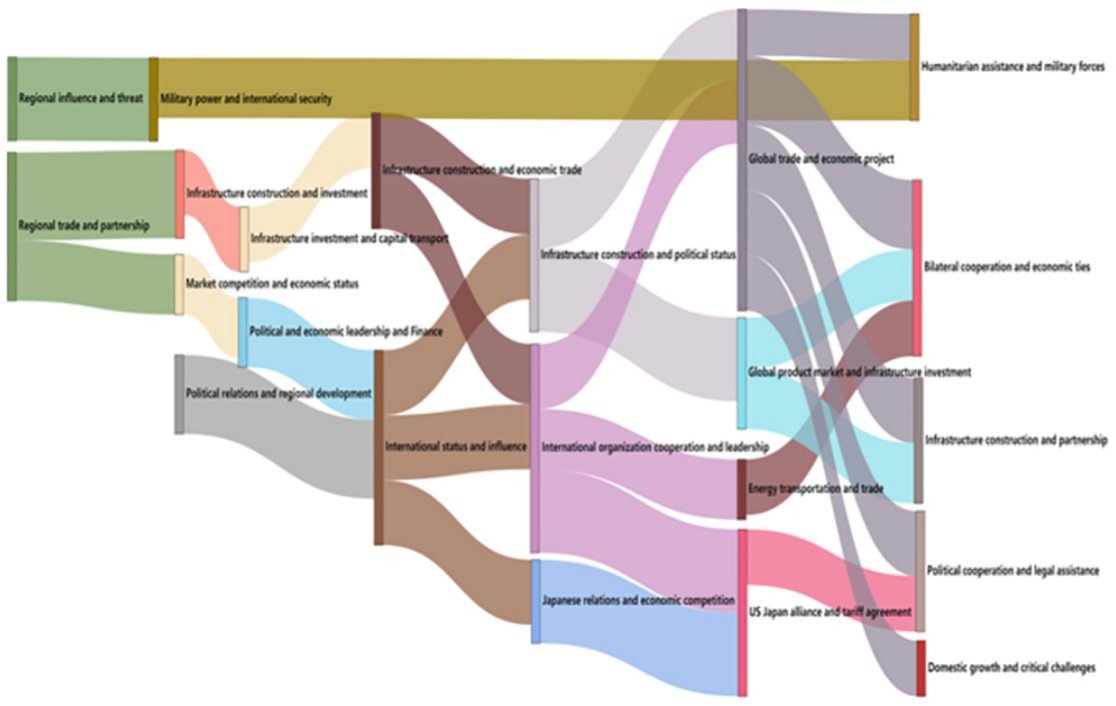
Evolution trend of think tanks’ research theme of BRI.

The evolution trend chart of BRI think tank reports, as shown in Fig 1, depicts the different themes of the same year using different node blocks, with each column representing the years from 2013 to 2020. The evolution of themes is represented by connecting lines. Due to the large amount of content in the figure, the image is not clear. Therefore, the chart of the evolution trend of BRI think tank reports is divided into three parts: 2013 to 2017, 2017 to 2018, and 2018 to 2020, for presentation.

**Fig 2:**
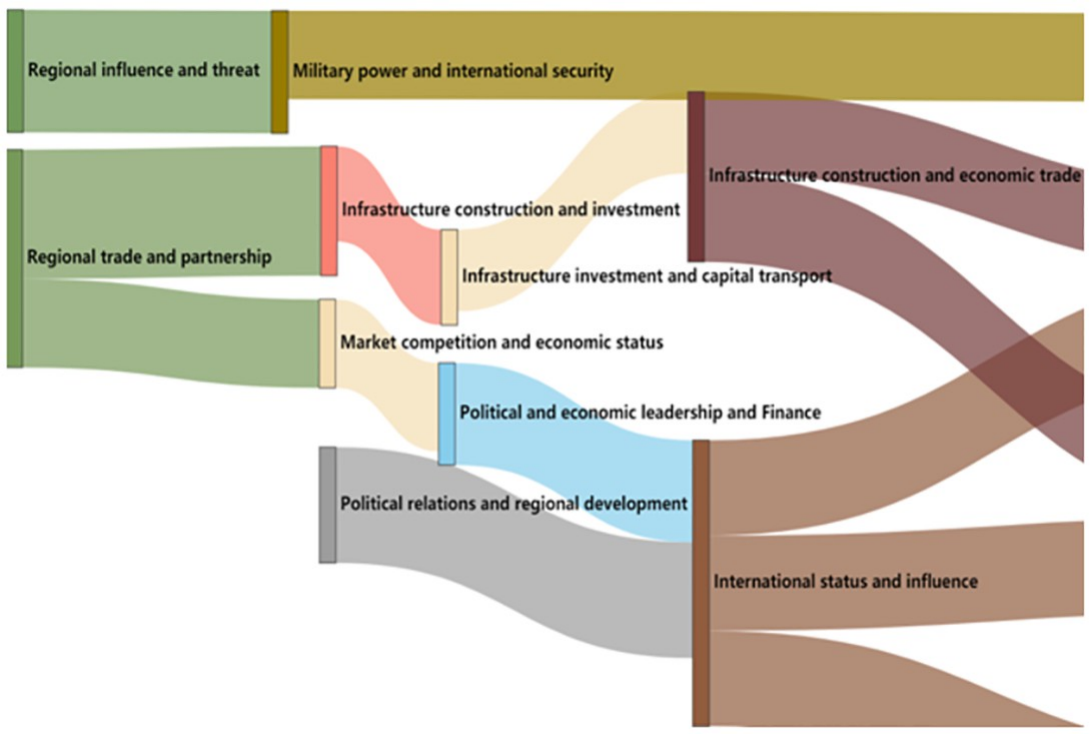
Evolution trend chart of think tanks’ research theme of” BRI (2013–2017).

From 2013 to 2017, the thematic evolution trend of BRI think tank reports is shown in Fig 2.

From Fig 2, it can be seen that in 2013, the research focus was centered on the political and economic aspects of the BRI. This included the study of regional trade, national relations, and the "sense of crisis" that the "BRI" brought to "Western" countries, among other topics. The research theme of regional influence and threats in 2013 evolved into the study of military competition and international security issues in the areas along the BRI in 2014. The emergence of this single theme was also influenced by the tense international security situation at that time, which led to a greater concentration of think tanks’ research on the BRI in the military field.

In 2015, the research theme continued to inherit and transform the research hotspots of 2013, and evolved into research topics in the field of economics such as infrastructure construction, market competition, and economic status. There is also a certain "enemy" meaning within it. In 2015, the research theme of political relations also emerged from the division of research topics in 2013, mainly focusing on the regional political impact of the BRI and the establishment of partnership issues.

In 2016, the research topics were mainly focused on the political and economic fields. One major theme revolved around the leadership position in the political and economic realms, specifically the changes in China’s national status and the status of other countries under the BRI. These changes stemmed from the economic competition and evolution of economic status in 2015. According to overseas think tanks, the proposal of the BRI by China has already expanded from economic competition to changes in political status. Another major theme is the continued investment in infrastructure under the BRI, which is also mentioned as a major advantage and focus point of our country’s initiative.

The theme inheritance of 2017 from 2016 is comprehensive, with a prominent focus on international status and influence. It not only continues the research hotspots of political and economic competition in 2016, but also inherits the national relations and regional development from 2015. This is because the enhancement of international influence is based on political relations and economic strength, naturally resulting in changes and comparisons in international status. Another theme is infrastructure construction and trade, which belongs to the economic field and continues the research themes of the previous years.

**Fig 3:**
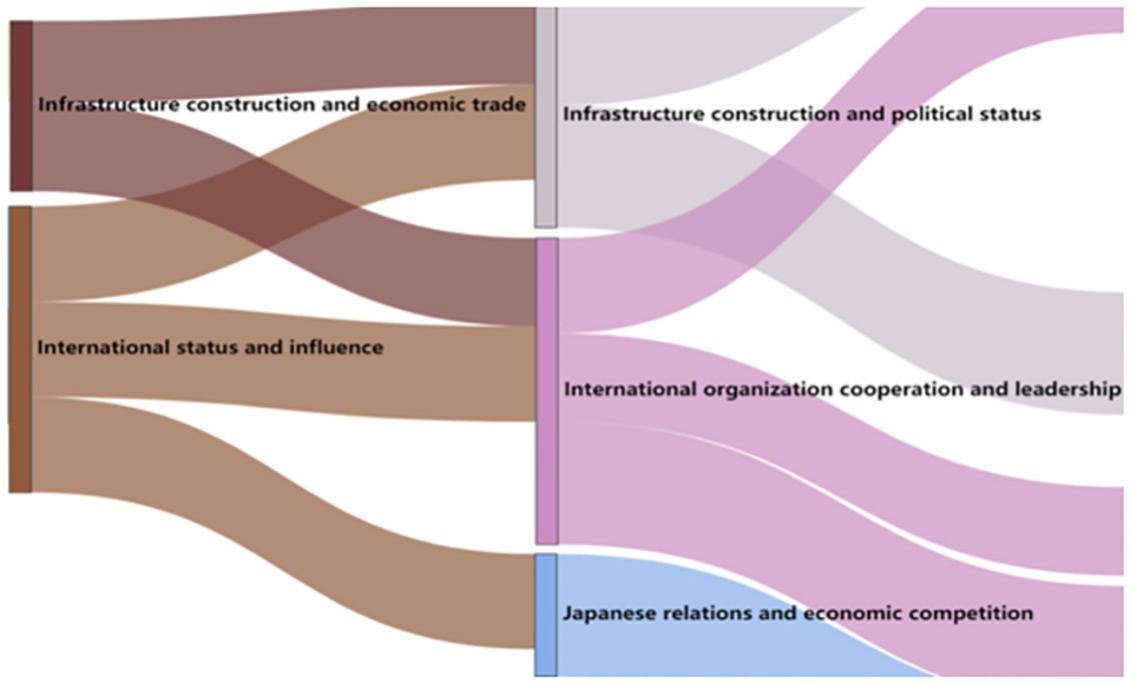
Evolution trend chart of think tanks’ research theme of the BRI (2017–2018).

From 2017 to 2018, the thematic evolution trend graph of the BRI think tank reports is shown in Fig 3.

According to Fig 3, it can be observed that the research topics in 2018 have started to increase. All three topics have evolved from international status and influence. Among them, the fusion of infrastructure construction and trade has led to the emergence of two themes: infrastructure construction and national status, and international organization cooperation and leadership. These themes also belong to the political and economic fields. The third theme is the competition between China and Japan in terms of their relationship and economic status. This theme inherits the research focus of international relations and expands into the economic field, highlighting the national relations and economic comparisons between China and Japan as the main countries.

**Fig 4:**
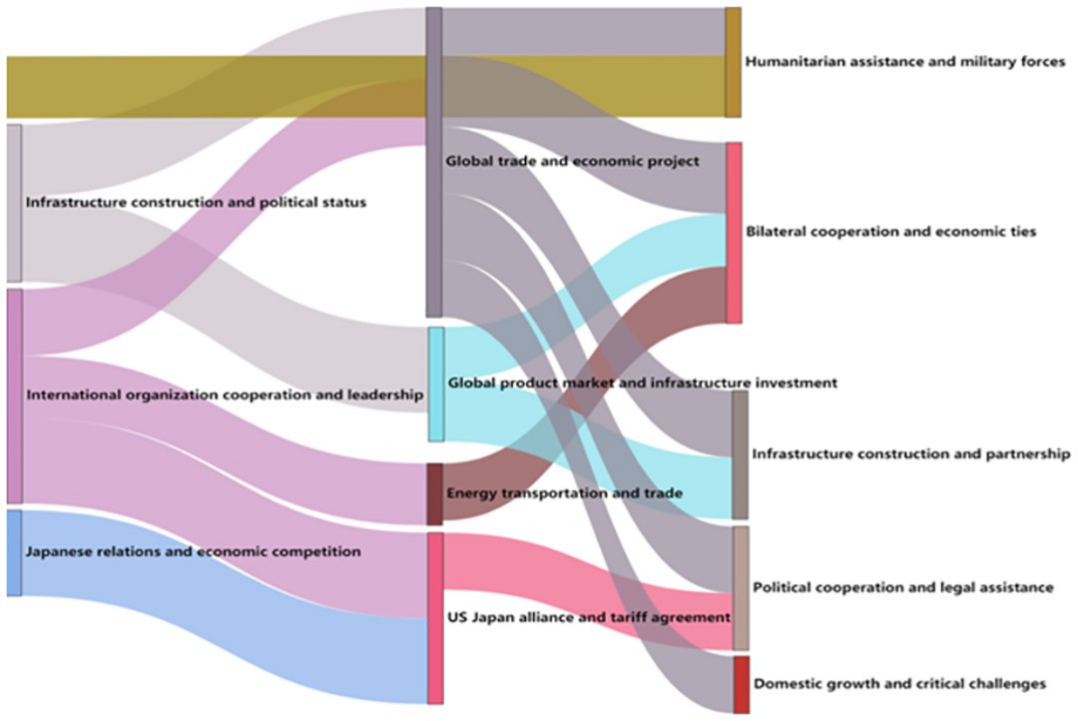
Evolution trend chart of think tanks’ research theme of the BRI (2018–2020).

From 2018 to 2020, the trend of the BRI think tank report’s thematic evolution is illustrated in Fig 4.

From Fig 4, it can be observed that the theme evolution during this stage is extremely rich. In the research topics of 2019, global trade and economic integration projects combined the research hotspots of infrastructure construction and international organization cooperation in 2018. Tracing back, it is the economic cooperation within the framework of international organizations that has promoted global trade under the BRI. Another theme that shows a trend of theme integration is the U.S.-Japan alliance and the tariff agreement. Based on the research on Sino-Japanese relations in 2018, the theme of the U.S.-Japan alliance emerged and combined with the BRI, the emergence of these themes actually corresponds to the dual nature of the BRI, which not only promotes cooperation and progress, but also gives rise to a multitude of complex international disputes and challenges. Think tanks are also studying the response measures of other countries [4].The research topic of the tariff agreement reflects the integration of the hot topic of international organization and also represents a more specific and detailed study of the BRI. Other research topics have evolved from infrastructure-related research, but the difference lies in the introduction of global product circulation and trade as research directions in infrastructure construction and investment. This characteristic reflects the trade issues related to the products under the BRI, which is closely related to the impact of the COVID-19 pandemic on the world. Another theme is energy transportation and trade, which has evolved from the previous year’s research on international organization cooperation and leadership. It also reflects the development of the BRI towards more important areas of cooperation projects, which has attracted more attention and in-depth research from think tanks.In 2020, the research hotspots have shown a closer connection with those of 2019 compared to other years. Additionally, there is a correlation between the research themes of different years and those of 2013. One such theme is humanitarian aid and military power, which is attributed to the facilitation of assistance to countries along the BRI, prompting overseas think tanks to explore national influence.

The reemergence of military-related topics in 2020 corresponds to the slightly unstable international security situation, thus leading to the resurgence of research in the military field since 2014. The two research themes of bilateral cooperation and economic ties, infrastructure construction and partnership belong to the continuous research on the BRI from a political and economic perspective. Political cooperation and legal assistance reflect the research at the new legal level [5], mainly focusing on the formulation of rules and guarantee issues in international cooperation of the BRI, and extending to the research on international trade and tariff agreements, due to the development and increased specialization of the BRI, as well as the diversity of participating members, various countries have begun to focus on reaching consensus on institutional rules. Another topic is domestic growth and key challenges, which is only inherited from the research topics of international trade in 2019, including not only the contribution and challenges of the BRI to China’s domestic development in global trade activities, but also the impact on the development level of countries mainly in the Western world. This bidirectional research hotspot reflects the substantive effect of the BRI in improving the development level of relevant countries, but also indicates that the research position of think tanks still carries the meaning of being challenging to the BRI, this also means that in the think tank’s view, the BRI essentially brings about a competition of international norms. The development of the BRI, as well as the reactions from other countries, can be seen as new challenges to these international norms [6]. And it also demonstrates that think tanks have their own positions in the essence of research on the BRI.

### Research summary

This article conducts a quantitative analysis of the research report on the BRI by the think tank, combining methods such as LDA2Vec topic modeling and word vector semantic similarity calculation from the perspectives of topic mining and topic evolution. The study finds that the think tank’s research on the BRI covers various aspects such as politics, economy, military, and law, with diverse research topics and rich topic evolution. The think tank’s analysis reflects the important position of the BRI strategy, as well as the attitude of cooperation and competition among countries. The next step should be to increase research in more areas such as environment, culture, and technology, and deepen the international positioning and related concept research of the BRI. This study has limitations, and future research will adopt more refined methods and expand research data. This article can provide a summary and summary of think tank research for BRI research institutions and personnel, promote the deepening of international BRI research, and provide reference for the BRI research.

## Supporting information

S1 Data (RAR)
